# From art to mental health: exploring the impact of a museum-based intervention on psychological well-being

**DOI:** 10.3389/fpsyg.2025.1591056

**Published:** 2025-11-14

**Authors:** Michela Nosè, Beatrice Compri, Doriana Cristofalo, Vanessa Carlon, Rada Kratchanova, Alessia Rodighiero, Carlotta Menegazzo, Federico Tedeschi, Giulia Turrini, Corrado Barbui

**Affiliations:** 1 WHO Collaborating Centre for Research and Training in Mental Health and Service Evaluation, Department of Neurosciences, Biomedicine, and Movement Sciences, Section of Psychiatry, University of Verona, Verona, Italy; 2 Palazzo Maffei Casa Museo, Fondazione Carlon, Verona, Italy

**Keywords:** psychological well-being, psychological distress, depressive symptoms, anxiety symptoms, art, museum

## Abstract

**Introduction:**

Engagement with arts and cultural activities has been increasingly recognized for its role in promoting mental health and well-being. This study evaluates the impact of a structured museum-based intervention on psychological well-being.

**Methods:**

A prospective hybrid type-1 non-randomized follow-up study with a pre-post design was conducted. Psychological distress, depressive symptoms, anxiety symptoms, and psychological well-being were assessed before and after a structured museum itinerary.

**Results:**

A total of 103 participants (82.5% female) completed pre- and post-intervention assessments. The intervention led to significant improvements across all psychological measures (*p* < 0.001), with the most notable effects observed in individuals aged 41–60. Additionally, between 89 and 98% of participants reported high acceptability, appropriateness, and feasibility of the itinerary.

**Discussion:**

The observed clinical improvements and high acceptability highlight the potential of museum-based interventions as an innovative and effective approach to mental health promotion. Future research should focus on scaling and replicating such interventions in diverse cultural and community settings, further integrating the arts into public health strategies.

## 1 Introduction

Mental health promotion interventions improve overall well-being and can be delivered in settings where people live, work, learn, and thrive. The World Health Organization (WHO) has defined the concept of health as “a state of complete physical, mental, and social well-being, which is more than the absence of mental disorders,” emphasizing not only the treatment of diseases but also the promotion of health and prevention of illnesses. Psychological well-being is an integral and essential part of an individual’s health. Recently, the European Commission recommended a comprehensive approach to mental health, and the results of a significant 2019 WHO study showed that the arts could potentially impact the prevention of diseases and the promotion of mental and physical health, as well as the management and treatment of diseases (Clift et al., 2019). The WHO Regional Office report analyzed evidence from over 900 global publications, concluding that engaging in artistic activities can benefit mental and physical health. The report examined the health benefits (through active or passive participation) across five major categories of arts: performing arts (music, dance, singing, theater, cinema); visual arts (crafts, design, painting, photography); literature (writing, reading, attending literary festivals); culture (visiting museums, galleries, concerts, theater); and online arts (animations, digital arts, etc.).

In recent years, research has increasingly suggested that museum visits could promote mental health and psychological well-being at both individual and community levels (Camic and Chatterjee, 2013; Nathalie and Legari, 2022; Thomson et al., 2018). Exposure to art has been shown to reduce symptoms of anxiety and depression while enhancing self-esteem, creativity, and intellectual engagement. Museums also provide restorative breaks from daily life, foster social cohesion, and reduce isolation, supporting both individual and community well-being. A systematic review by Bosman et al. (2021) highlighted the potential of art therapy to decrease symptoms of anxiety and depression and improve quality of life. Ioannides (2016) discussed how art therapists can transform museum settings into “therapeutic holding environments”, helping individuals address physical, emotional, and mental challenges through creative expression. Likewise, engagement with art activates brain regions associated with positive emotions, promoting emotional regulation, stress reduction, and subjective enjoyment (Mastandrea et al., 2019). Museums and art galleries have been identified as effective settings for public health interventions, combining cultural experiences with targeted programs that support personal growth, including increased self-esteem, a stronger sense of identity, reduced social isolation, and decreased anxiety (Chatterjee, 2016). Early research also highlights the restorative effects of museums, showing that they reduce mental fatigue and provide spaces for reflection and engagement (Kaplan et al., 1993). These beneficial effects have been observed across diverse populations, regardless of age, cultural background, or socioeconomic status, and have been further documented in national and international reports emphasizing museums’ value in promoting well-being (Froggett et al., 2011; Dodd and Jones, 2014; Lackoi et al., 2016; Falk et al., 2023). Despite promising evidence, most of these studies remain observational or descriptive, with limited experimental research. Moreover, although there is a summary of studies describing the benefits of museum activities in healthcare contexts, there are no studies covering psychological symptoms as a benefit of museum visitation for the general public. In light of this, it seems particularly important to investigate whether structured multi-day museum experiences can positively impact psychological symptoms and overall well-being in the general population.

Palazzo Maffei Fondazione Carlon in Verona was born from the private collection of Luigi Carlon, which, over more than 60 years of passion for art, has collected over 650 works with an eclectic taste, now housed in the seventeenth-century Palazzo Maffei’s 28 rooms. The exhibition itinerary is surprising because it brings together paintings, sculptures, drawings and an important selection of objects of applied art distinguished by an everlasting eclecticism and designed as a walk-through art history, from Greco-Roman archeology to contemporary site-specific installations.

Data emerging from the international literature and the collaboration between Palazzo Maffei and the WHO Collaborating Center for Mental Health Research at the University of Verona provide the framework for the MINERVA project (Museum, Innovation, Neurosciences: Effects of and Reactions to the Value of Art). MINERVA is a multidisciplinary initiative specifically developed to investigate the psychological and clinical effects of museum-based cultural itineraries. The project has been developed to create a cultural path within a museum setting in response to the growing need to support the psychological well-being of people. The main aims of the study were: (1) to assess the impact of artistic experiences, particularly museum experiences, in enhancing psychological well-being and reducing anxiety-depressive symptoms and distress, and (2) evaluating their implementability. The secondary objectives of the project were to evaluate the factors associated with clinical improvement resulting from the artistic experiences, as well as to assess whether there are groups of people who could benefit most from the museum experiences.

## 2 Materials and methods

### 2.1 Study design

Between June and November 2024, we conducted a prospective hybrid type-1 non-randomized follow-up study, as described by Curran et al. (2012), with a pre-post design, where the first assessment (T1) was completed before the museum itinerary, while the second assessment was conducted after the itinerary, at the end of the third session (T2).

Participation in the project was organized in three consecutive groups during the study period. All groups followed the same structured museum itinerary. Adult participants were recruited through Palazzo Maffei’s communication channels, including public announcements on the institutional website, flyers, newsletters, word-of-mouth, emails, and social networks. For each phase, a maximum of 40 participants was set based on the order of registration, with no additional inclusion or exclusion criteria.

### 2.2 Data collection and study procedures

Before enrollment, participants were informed online about the nature of the study and the proposed museum itinerary. They were given the option to choose whether to participate or decline. It was clarified that participation was voluntary, and they could withdraw from the study anytime. An online informed consent was electronically signed by all participants who decided to participate at the beginning of the first sessions. After that, they accessed LimeSurvey, the software used in this study, to collect online pre- and post-museum itinerary data. The pre-and post-assessments consisted of the collection of ad-hoc sociodemographic information (age, gender, educational qualification, marital status, employment status), and information on psychological condition, collected through four self-administered questionnaires assessing psychological distress as measured with the Kessler-10 (K-10; Kessler et al., 2002), depressive symptoms as measured with the nine-item Patient Health Questionnaire depression scale (PHQ-9; Kroenke et al., 2001), anxiety symptoms with the seven-item Generalized Anxiety Disorder scale (GAD-7; Spitzer et al., 2006), and psychological well-being with the WHO-5 Wellbeing Index (WHO-5; Sischka et al., 2020). In addition, at post-intervention, the acceptability, appropriateness and feasibility of the intervention were assessed using the adapted versions of the Acceptability of Intervention Measure (AIM), the Intervention Appropriateness Measure (IAM), and the Feasibility of Intervention Measure (FIM; Weiner et al., 2017). Participants completed the first assessment (T1) before the museum itinerary, while the post-assessment was completed after the end of the itinerary (T2). The data collected through LimeSurvey were stored safely in a password-protected CSV file for further analysis.

The study has been reviewed and approved by the Institutional Review Board (IRB) of the University of Verona (registration number #13/2024).

### 2.3 Inclusion criteria

Participants were at least 18 years old. Only participants who electronically signed the informed consent form before entering the study were enrolled, and only participants who had guaranteed attendance at all three sessions of the museum itinerary were included. No exclusion criteria were applied.

### 2.4 Intervention: museum itinerary

The museum collection has a chronological and thematical order. In the first section of the Museum, characterized by the view over Piazza delle Erbe in the heart of Verona, a dialog has been created, where a piece of modern art unexpectedly breaks the thematically arranged classical works. In the second section, dedicated to the 20th century and contemporary art, a veritable gallery space has been created, where numerous masterpieces stand out, and the passion for Futurism and Metaphysical art can be seen through the works of great artists. On the second floor, an additional eight exhibition rooms and a project room offer a path that invites reflection, thanks to installations by contemporary artists.

The museum itinerary consisted of a structured cultural path in three weekly guided visits, each lasting less than an hour. An art historian from the Museum, informed about the study objectives, guided participants to engage with the exhibited masterpieces, encouraging observation, reflection, and discussion. This active participation was designed to promote attention, emotional engagement, and cognitive stimulation. The structured itinerary exposed participants to a diverse range of artistic experiences, supporting both emotional and cognitive wellbeing. Each guided visit differed from the previous one in the themes examined, focusing participants’ attention and curiosity on the varied facets of the Collection. The objective of each visit was to convey the dual essence of the exhibition through a dialog between ancient and modern, the curatorial approach, the museography design, and the variety of perspectives with which to interpret a single work. The museum itinerary, organized during the three visits, was structured as follows:

#### 2.4.1 1st visit. Ancient and modern: a continuous dialog

The Palazzo Maffei Collection presents a dual essence due to the eclecticism of a collection that has grown without chronological or genre limits, driven by private collecting passion. The first session aims to welcome and guide participants through a kaleidoscopic path between ancient and contemporary, translating the dialogs that arise from the juxtaposition of different eras and styles. By approaching and decoding the principal works and their exhibition rationale, participants will gain an understanding of the Collection, from masterpieces to curiosities.

#### 2.4.2 2nd visit. Science hidden in art

The “Science Hidden in Art” session aims to reveal the close connection between scientific and humanistic realities. Not opposites but cross-disciplinary fields linked by mutual interdependence. Through the Palazzo Maffei Collection, participants will spark an interest in both worlds, demonstrating how scientific research often lies behind artistic aesthetics.

#### 2.4.3 3rd visit. Art and psychological well-being: a connection with the works

The final session offers a journey to discover art’s more profound and spiritual value, revealing how artistic potential, beyond its aesthetic pleasure, transmits profound stimuli that benefit psychological well-being. According to Kandinsky’s vision, the viewer must learn to see the image as a graphic representation of an emotional state. The itinerary focuses on the relationship between individual and collective well-being and the Museum’s role.

### 2.5 Clinical measures

#### 2.5.1 Psychological distress

The K-10 is a 10-item self-report questionnaire to screen broadly for psychological distress experienced in the past 30 days (Kessler et al., 2002). Its items are rated on a five-point Likert scale ranging from “none of the time” to “all of the time.” The K-10 has good psychometric properties (Wojujutari and Idemudia, 2024).

#### 2.5.2 Depression and anxiety symptoms

The Patient Health Questionnaire - Anxiety and Depression Scale (PHQ-ADS; Kroenke et al., 2016) is a 16-item self-reported instrument that combines PHQ-9 (Kroenke et al., 2001) and GAD-7 (Spitzer et al., 2006) into a composite measure of depression and anxiety. Respondents are asked how much each symptom has bothered them over the past 2 weeks, with response options of “*not at all*,” “*several days*,” “*more than half the days*,” and “*nearly every day*,” scored as 0, 1, 2, and 3. The scale can range from 0 to 27 in the case of PHQ-9 and from 0 to 21 in the case of GAD-7, with higher scores indicating higher levels of depression and anxiety symptoms.

#### 2.5.3 Psychological wellbeing

The WHO-5 is a 5-item questionnaire measuring current psychological well-being and quality of life rather than psychopathology (Sischka et al., 2020). Scores range from 0 to 25, and the scale has demonstrated sensitivity to change in well-being. It is available in numerous languages and was administered in Italian to all participants in this study (Sischka et al., 2020).

All the measures were adapted to be fulfilled in LimeSurvey.

### 2.6 Implementability measures

Implementability measures include acceptability, appropriateness and feasibility. Quantitative data on participants’ points of view on the implementability of museum itineraries were gathered. For this purpose, adapted versions of the Acceptability of Intervention Measure (AIM), the Intervention Appropriateness Measure (IAM), and the Feasibility of Intervention Measure (FIM) were administered during the T2 assessment (Weiner et al., 2017). In particular, four items were selected: two for acceptability (“*the museum itinerary has satisfied me*” and “*the museum itinerary has interested me*”), one for appropriateness (“*the museum itinerary seemed appropriate for my needs*”) and one for feasibility (“*the museum itinerary seemed easy for me to use*”). Scale values range from 1 to 5. Norms and cut-off scores for interpretation are not yet available; however, higher scores indicate greater acceptability, appropriateness, or feasibility.

### 2.7 Statistical analysis

Descriptive statistics were used for demographic and clinical variables at both time points. Continuous variables were expressed as means and standard deviations, while categorical variables were expressed as absolute numbers and percentages. The following sociodemographic variables of interest were collected: age, gender, educational level, marital status, employment status and whether the person had children. Clinical scales were both considered as continuous and grouped into clinically meaningful categories according to established cut-off scores. For the K-10, psychological distress was classified as absence (0–15) vs. presence (16–50). Anxiety (GAD-7) was classified as absent or minimal (0–4), mild (5–9), moderate (10–14), or severe (15–21). Depression (PHQ-9) was classified as absent (0–4), subthreshold (5–9), mild (10–14), moderate (15–19), or severe (20–27). Psychological well-being (WHO-5) was categorized as high (17–25), medium (9–16), or low (0–8).

For each clinical outcome, means and standard deviations were calculated for both baseline and post-treatment values. The presence of change between baseline and follow-up was assessed through the test on the median of differences between matched pairs (Ridgman, 1990), to allow for non-normality of the pre-post difference distribution. In the case of the implementability outcomes, the percentage of participants replying either “agree” or “completely agree” was calculated for each item and considered as an indicator of satisfaction.

Regression analyses were performed, having as outcomes the total scores of PHQ-9, GAD-7, K-10 and WHO-5, and, as predictors, sociodemographic variables (age, gender, having a bachelor degree, being in a stable relationship, being employed, being a parent), controlling for the baseline value of the outcome in order to interpret results in terms of conditional association with change. The statistical significance of the three parameters related to age was always assessed jointly, to make results independent from the choice of the reference category.

A global model with Seemingly Unrelated Regression (SUR) equations (Zellner, 1962) was performed, in its modification to allow for unbalanced data proposed in Baum and Schaffer (2009) through the Stata ‘suregub’ command. This allowed us to evaluate the overall statistical significance of the regressions by taking the between-outcome correlation of residuals into account. In case such global statistical significance of the coefficients associated to the predictors, we proceeded to perform the four single regressions. A global test across the four regressions was also performed for each predictor separately, and only in case of its statistical significance results from single regressions were considered.

Statistical analyses were conducted using Stata 18 (Statacorp, 2023).

## 3 Results

### 3.1 Sociodemographic and clinical characteristics at baseline

Of the 128 participants who expressed interest in the project, 103 were included in the analyses as they attended all three museum visits and completed the questionnaires at T1 and T2. The recruited 103 participants comprised 82.5% females, with an average age of 53. More than half had a master’s degree (47% had it as the highest title, and 12% had a PhD, master or residency). Among the sample, 43% were married, 63% had children, and were in various employment situations. Overall, it was a heterogeneous sample, representing diverse sociodemographic characteristics (Table 1). As reported in Table 2, at baseline (T1), the sample, on average, reported significant psychological distress in 67% of cases before the start of the museum itinerary, with mild anxiety in almost half of the cases and depressive symptoms of varying severity, coupled with medium to low levels of psychological well-being for almost two thirds of the sample.

**Table 1:** Sociodemographic characteristics (*n* = 103).

Gender	*N (%)*
Female	85 (82.5)
Male	18 (17.5)
Age	*N (%)*
Mean (*SD*)	52.85 (15.20)
18–40	21 (20.4)
41–60	46 (44.7)
>60	36 (34.9)
Educational level	*N (%)*
Professional qualification	5 (4.9)
High school diploma	33 (32.0)
Bachelor’s Degree	5 (4.9)
Master’s Degree	48 (46.6)
Residency/Post-graduate/PhD	12 (11.7)
Marital status	*N (%)*
Single	17 (16.5)
Married	44 (42.7)
Stable Relationship/Cohabiting	19 (18.4)
Separated	8 (7.8)
Divorced	7 (6.8)
Widowed	8 (7.8)
Children	*N (%)*
Yes	65 (63.1)
No	38 (36.9)
Employment status	*N (%)*
Student/PhD/Resident	8 (7.8)
Permanent Contract	30 (29.1)
Fixed-Term Contract	3 (2.9)
Self-Employed/Freelancer	14 (13.6)
Retired	34 (33.0)
Other	14 (13.6)

**Table 2:** Clinical variables before (T1) and after (T2) the museum itinerary (*n* = 103).

	T1		T2		*p-*value
K-10	*N*	*%*	*N*	*%*	
Absence of psychological distress	34	33.0	45	43.7	**<0.001**
Presence of psychological distress	69	67.0	58	56.3	
Total (*N*)	103		103		
Mean (*SD*)	18.93 (5.78)		16.92 (5.01)		
GAD-7	*N*	*%*			
Absent or Minimal Anxiety	42	40.8	53	51.5	**<0.001**
Mild Anxiety	47	45.6	43	41.7	
Moderate Anxiety	11	10.7	5	4.9	
Severe Anxiety	3	2.9	2	1.9	
Total (*N*)	103		103		
Mean (*SD*)	5.73 (3.66)		4.57 (3.28)		
PHQ-9	*N*	*%*			
Absence of depression	24	23.5	44	42.7	**<0.001**
Subthreshold depression	48	47.1	44	42.7	
Mild major depression	21	20.6	10	9.7	
Moderate major depression	6	5.9	2	1.9	
Severe major depression	3	2.9	3	2.9	
Total (*N*)	102		103		
Mean (*SD*)	6.22 (4.41)		4.77 (3.65)		
WHO-5	*N*	*%*			
High psychological well-being (17–25)	35	34.3	52	50.5	**<0.001**
Medium psychological well-being (9–16)	50	49.0	43	41.7	
Low psychological well-being (1–8)	17	16.7	8	7.8	
Total (*N*)	102		103		
Mean (*SD*)	13.88 (5.47)		15.88 (5.01)		

Bold values indicate statistically significant differences.

### 3.2 Effectiveness of museum itinerary

After completing the three scheduled visits (T2), we observed significant improvements across all measured outcomes, with a reduction in psychological symptoms, anxiety, and depressive symptoms and an increase in general psychological well-being. The mean score for psychological distress (K-10) decreased from 18.93 (*SD* = 5.78) at baseline to 16.92 (*SD* = 5.01) at post-intervention, indicating a significant reduction in distress levels (*p* < 0.001). At T1, 67% of participants showed signs of psychological distress, which decreased to 56% at T2. Mean anxiety scores (GAD-7) decreased from 5.73 (*SD* = 3.66) at baseline to 4.57 (*SD* = 3.28) at post-intervention (*p* < 0.001). The proportion of participants with moderate or severe anxiety halved from 13.6% at T1 to 6.8% at T2. Depression scores (PHQ-9) also improved, with a mean score reduction from 6.22 (*SD* = 4.41) at baseline to 4.77 (*SD* = 3.65) at post-intervention (*p* < 0.001). The proportion of participants with moderate to severe depression dropped from 8.8 to 4.8%. The mean score for psychological well-being (WHO-5) increased from 13.88 (*SD* = 5.47) at T1 to 15.88 (*SD* = 5.01) at T2 (*p* < 0.001), reflecting a significant enhancement in students’ well-being. The percentage of students reporting high well-being increased from 34% at T1 to 50% at T2 (see Table 2).

### 3.3 Implementability of museum itinerary

In relation to the implementability outcomes, the itinerary was highly appreciated by the participants. Ninety-seven per cent of participants either agreed or completely agreed that the museum itinerary satisfied them, 98% felt it was interesting, and 89% appropriate for their needs. Finally, 97% agreed or completely agreed that the museum itinerary was accessible and easy to use (Table 3).

**Table 3:** Measures of implementability (*n* = 103).

	Completely disagree	Disagree	Neither agree nor disagree	Agree	Completely agree
The museum route has satisfied me	0	0	3 (2.9)	19 (18.4)	81 (78.6)
The museum route has interested me	0	0	2 (1.9)	17 (16.5)	84 (81.6)
The museum route seemed appropriate for my needs	0	2 (1.9)	9 (8.7)	33 (32.0)	59 (57.3)
The museum route seemed accessible to me	0	0	3 (2.9)	29 (28.2)	71 (68.9)

### 3.4 Factors associated with clinical outcomes

The predictors demonstrated global statistical significance across the four regression models (*p* = 0.023). Among individual predictors, age emerged as the only variable with global statistical significance (*p* < 0.001), while gender (*p* = 0.267), educational attainment (bachelor’s degree; *p* = 0.271), relationship status (*p* = 0.291), employment status (*p* = 0.258), and parental status (*p* = 0.838) did not exhibit significant associations.

In the final regressions, age turned out as statistically significant for K-10 (*p* < 0.001) and GAD-7 (*p* = 0.043). Individuals aged 41–60 years conditionally showed substantial clinical improvements compared to the 18–24 age group, with average reductions of 5.33 points in K10 and 2.66 points in GAD-7 scores, after adjusting for other predictors and their respective baseline outcomes.

Detailed regression results are presented in Table 4.

**Table 4:** Regression Analyses of WHO-5, PHQ, ADS, and K-10 on age groups (*n* = 103).

Age group	WHO-5	CI	*p-*value	PHQ-9	CI	*p-*value	GAD-7	CI	*p-*value	K-10	CI	*p-*value
26–40 vs. 18–25	0.285	−3.502, 4.071	0.159	0.891	−1.884, 3.667	0.162	−1.187	−3.529, 1.154	0.043	−2.156	−5.489, 1.177	<0.001
41–60 vs. 18–25	2.167	−1.423, 5.757		−1.306	−3.960, 1.347		−2.658	−4.869, −0.448		−5.426	−8.631, −2.221	
61 + vs. 18–25	−0.319	−4.318, 3.679		−0.251	−3.204, 2.702		−1.274	−3.726, 1.178		−1.869	−5.431, 1.693	

Regression analyses controlling for gender, marital status, educational level, employment status.

## 4 Discussion

This study aimed to evaluate the clinical benefits and implementability of a structured museum itinerary in enhancing psychological well-being, reducing anxiety-depressive symptoms, and distress. The findings support the potential of museum-based interventions as an innovative and practical approach to mental health promotion. The results demonstrate substantial improvements across all clinical outcomes, including psychological distress, anxiety, depressive symptoms, and psychological well-being. The significant reduction in psychological distress reflects a meaningful decrease among participants, aligning with previous literature highlighting the therapeutic role of arts and cultural activities in fostering emotional regulation and stress reduction. Furthermore, the reduction in anxiety and depressive symptoms highlights the potential of the museum itinerary as a complementary approach for individuals facing mild to moderate psychological difficulties. Enhancing psychological well-being further underscores the itinerary’s ability to foster positive mental states. The percentage of participants reporting high well-being increased from 34 to 50%, highlighting the intervention’s success in promoting quality of life and overall mental health. These findings are particularly significant given the diverse demographic composition of the sample, which ranged widely in age (18–85 years), educational background, and employment status, suggesting that structured cultural experiences can transcend demographic differences to deliver meaningful benefits. However, the regression analyses indicated that age may play a role in shaping clinical outcomes, with the most pronounced improvements in psychological distress and anxiety symptoms associated to being aged 41–60. The implementability measures demonstrated the intervention’s high acceptability, appropriateness, and feasibility. Nearly all participants (97–98%) agreed or strongly agreed that the itinerary was satisfying and interesting, while 89% found it appropriate for their needs and accessible. These results indicate that the museum itinerary was well-received and logistically practical for the target population, likely attributable to the dynamic and interactive nature of the guided visits, which emphasized thematic diversity and encouraged active involvement with the artworks. The structured format of the itinerary, featuring three weekly visits with distinct themes, allowed participants to explore the intersection of art, science, and psychological well-being, facilitating more profound connections with the exhibited works and enhancing the overall impact of the experience. The findings of this study align with global recommendations for integrating arts and cultural activities into mental health strategies. The significant improvements in mental health outcomes and the high acceptability of the intervention suggest that museum-based programs can serve as a scalable and cost-effective approach to promoting psychological well-being. The results reinforce the value of interdisciplinary collaborations between cultural institutions and mental health researchers in developing innovative interventions. Given the increasing prevalence of mental health challenges worldwide, particularly in the aftermath of the COVID-19 pandemic, there is an urgent need for accessible and non-stigmatizing interventions. Museum experiences, which combine aesthetic, emotional, and social dimensions, provide a unique opportunity to address this need, complement traditional mental health care, and reach broader populations.

Our findings align with growing evidence that museums can play a significant role in supporting mental health and psychological well-being. For example, Beauchet et al. (2022) showed that a three-month cycle of art-based museum activities improved well-being and social connectedness in older adults, while a later study demonstrated that even a single physician-prescribed museum visit could positively impact patients’ quality of life (Beauchet et al., 2025). This intervention focused on free exploration of art rather than participatory activities. While both forms of engagement have demonstrated mental health benefits, they may rely on different mechanisms, such as expression and social interaction in participatory activities vs. aesthetic appreciation and contemplation in receptive ones. Additional evidence comes from museum-based participatory arts programs, such as the study by Goodman-Casanova et al. (2023), which demonstrated benefits for mental health recovery and social support among service users. Complementary findings are reported in the ARTEMIS project, which showed that structured museum interventions promoted emotional well-being and improved mood and quality of life in people with dementia (Schall et al., 2018). From a theoretical perspective, our results resonate with Kaplan’s Attention Restoration Theory (Kaplan et al., 1993), which posits that environments possessing qualities such as being away, extent, fascination, and compatibility, especially when combined with aesthetic engagement, can restore directed attention and promote reflection, thereby supporting psychological well-being. Our findings also align with Seligman’s PERMA model of well-being (Seligman, 2011), particularly the components of positive emotion, engagement, and meaning. These frameworks help explain why structured museum experiences may alleviate anxiety-depressive symptoms and foster psychological well-being. A recent systematic review further confirmed that museums can enhance visitors’ well-being when experiences are designed to be restorative, participatory, comprehensible, appealing, and sustainable (Šveb Dragija and Jelinčić, 2022). However, the review also emphasized that most studies remain descriptive, with limited experimental evidence and few investigations in general populations. While much of the existing literature has focused on social, emotional, or participatory dimensions, the unique contribution of our study lies in its focus on clinical symptoms within the general population, thereby addressing a critical gap in the evidence base.

### 4.1 Limitations and future directions

Despite the promising findings, this study has several limitations. The non-randomized design and reliance on self-reported measures may introduce biases, such as social desirability or recall bias: randomized controlled trials are needed to evaluate the effectiveness of this kind of intervention and to control bias. Since the study lacked a randomized control group, it is challenging to disentangle the actual effects of the museum itinerary from natural fluctuations or regression to the mean. Future studies could address this by incorporating repeated baseline measurements or a randomized control design to isolate the intervention’s impact better. Additionally, one of the key limitations of this study is that data collection was restricted to individuals who completed the entire museum itinerary. As a result, we lack information, particularly regarding the satisfaction and perceptions of those who expressed interest but did not participate fully. This information would be valuable for understanding potential barriers and facilitators to participation and improving the overall implementability of such interventions. Future research should aim to include data from all individuals who demonstrate interest in the program, even if they do not complete it, to gain a more comprehensive understanding of the intervention’s appeal and accessibility. Regarding the generalizability of the findings, the predominance of female participants (82.5%) and the relatively high educational levels of the sample may limit it. Future studies should aim to include more diverse and representative samples to explore potential differences in the intervention’s impact across various demographic groups. Additionally, research could focus on tailored interventions for specific age groups, particularly middle-aged adults, to determine the most effective strategies for engaging these populations and enhancing the therapeutic benefits of museum experiences. Finally, while the study assessed short-term outcomes, the long-term sustainability of the observed benefits remains unclear. Longitudinal studies are needed to evaluate whether psychological well-being and symptomatology improvements persist. Further research could also investigate the mechanisms underlying the intervention’s effects, such as the role of social interaction, aesthetic appreciation, or cognitive engagement.

## 5 Conclusion

This study highlights the potential of structured museum itineraries as beneficial and implementable interventions for promoting mental health and psychological well-being. The significant improvements in clinical outcomes and the high acceptability of the intervention underscore the value of integrating cultural experiences into mental health promotion strategies. By leveraging the unique strengths of museums as spaces for learning, reflection, and emotional connection, this approach offers a promising avenue for enhancing mental health in diverse populations. Future efforts should focus on scaling and replicating such interventions in other cultural and community settings, further advancing the role of arts in public health.

## Data Availability

The raw data supporting the conclusions of this article will be made available by the authors, without undue reservation.
